# Physician preferences for nonmetastatic castration-resistant prostate cancer treatment in China

**DOI:** 10.3389/fonc.2024.1382678

**Published:** 2024-05-21

**Authors:** Yu Fan, Xuanjun Guo, Davide Campobasso, Zhisong He

**Affiliations:** ^1^ Department of Urology, Peking University First Hospital, Beijing, China; ^2^ Institute of Urology, Peking University, Beijing, China; ^3^ National Urological Cancer Center, Beijing, China; ^4^ Division of Urology, Azienda Ospedaliero-Universitaria of Parma, Parma, Italy; ^5^ Prostate Cancer Unit, Azienda Ospedaliero-Universitaria of Parma, Parma, Italy

**Keywords:** nonmetastatic castration-resistant prostate cancer (nmCRPC), second-generation androgen receptor inhibitors (SGARIs), choice behavior, quality of life, physicians

## Abstract

**Introduction:**

The treatment preferences of Chinese physicians who treat nonmetastatic castration-resistant prostate cancer (nmCRPC) and how they weigh the benefits and risks of nmCRPC treatment are still unknown. This study aimed to evaluate Chinese physicians’ benefit–risk treatment preferences for nmCRPC and assist in setting nmCRPC treatment goals.

**Methods:**

A paper-based discrete choice experiment (DCE) survey was administered to 80 nmCRPC-treating physicians. DCE responses were analyzed to produce the preference weight and the relative importance score for each attribute level. The marginal rate of substitution (MRS) was used to quantify the amount of overall survival (OS) physicians were willing to trade for a reduction in treatment-related adverse events (AEs). We further conducted the exploratory analysis, stratifying physicians from 5 perspectives into different subgroups and examining the treatment preferences and OS trade-off in each subgroup.

**Results:**

In terms of efficacy attributes, physicians placed greater emphasis on OS than time to pain progression. With regard to safety attributes, serious fracture was perceived as the most important AE by physicians, followed by serious fall, cognitive problems, skin rash, and fatigue. In the exploratory analysis, we found generally that physicians with less clinical practice experience and those from more economically developed regions placed more emphasis on AEs and were willing to give up more of their patients’ OS to reduce the risk of AEs.

**Conclusion:**

Physicians from mainland China value the importance of minimizing treatment-related AEs when considering different treatment options for patients with nmCRPC, and they are willing to trade a substantial amount of OS to avoid AEs.

## 1 Introduction

Nonmetastatic castration-resistant prostate cancer (nmCRPC) is an intermediate stage of prostate cancer (PC), which is characterized by persistently elevated prostate-specific antigen (PSA) but without detectable radiological evidence showing distant metastasis even after androgen deprivation therapy (ADT) (1). Patients with nmCRPC are in a crucial period of disease before metastasis (2, 3). Therefore, in case of progression, it is necessary to adopt therapeutic interventions (4), and notably, patients with nmCRPC are often asymptomatic (5). Therefore, both delaying metastatic progression and reducing treatment-related adverse events (AEs) are significant therapeutic goals for patients with nmCRPC (6–8).

Until recently, treatment options for nmCRPC have included ADT with first-generation androgen receptor inhibitors (ARIs) (9), as well as novel second-generation androgen receptor inhibitors (SGARIs), including apalutamide, enzalutamide, and darolutamide. Large-scale randomized controlled phase III clinical trials have demonstrated that SGARIs remarkably improve the metastasis-free survival (MFS) and overall survival (OS) in patients with nmCRPC (10, 11). However, they may also increase the risk of AEs, which may adversely affect the health-related quality of life (HRQoL) of patients with nmCRPC (12, 13). These 3 SGARIs vary in their AEs (10, 14–16). For example, the reported rates of fatigue are 30% for apalutamide, 33% for enzalutamide, and 12.1% for darolutamide, while the rates of rash and fall range from 2.9% to 24% and from 4.2% to 16%, respectively (17–19). Compared to the other 2 SGARIs, darolutamide has a lower propensity for blood brain–barrier penetration and thus can reduce the incidence of central nervous system–associated AEs and fatigue (20, 21).

nmCRPC physicians, patients, and caregivers are often faced with a tradeoff between efficacy and AEs when choosing the treatment option. Patients’ treatment choices are often influenced by physicians’ professional advice. Therefore, understanding physicians’ preferences for nmCRPC treatment and how they weigh treatment benefits and risks will help us to better understand the value they place on the minimization of AEs and guide treatment decisions. There is thus far a lack of research on physician treatment preferences for patients with nmCRPC in mainland China. In this study, we therefore used a discrete choice experiment (DCE) approach to assess physicians’ benefit–risk preferences for treatment attributes and to further explore physicians’ tradeoffs between treatment efficacy and AEs.

## 2 Methods

### 2.1 Study design and setting

This was a paper-based questionnaire study with 5 main components, including designing a physician questionnaire, validating the questionnaire through 2 clinical expert interviews, revising and finalizing the questionnaire, recruiting physicians with experience in treating patients with nmCRPC from urology or oncology to conduct the offline questionnaire, analyzing the data, and reporting the results. The study was based on a survey study of patients and caregivers with nmCRPC conducted in the United States, and the DCE model of the study was designed and modified according to the Chinese context (13).

**Figure 1:**
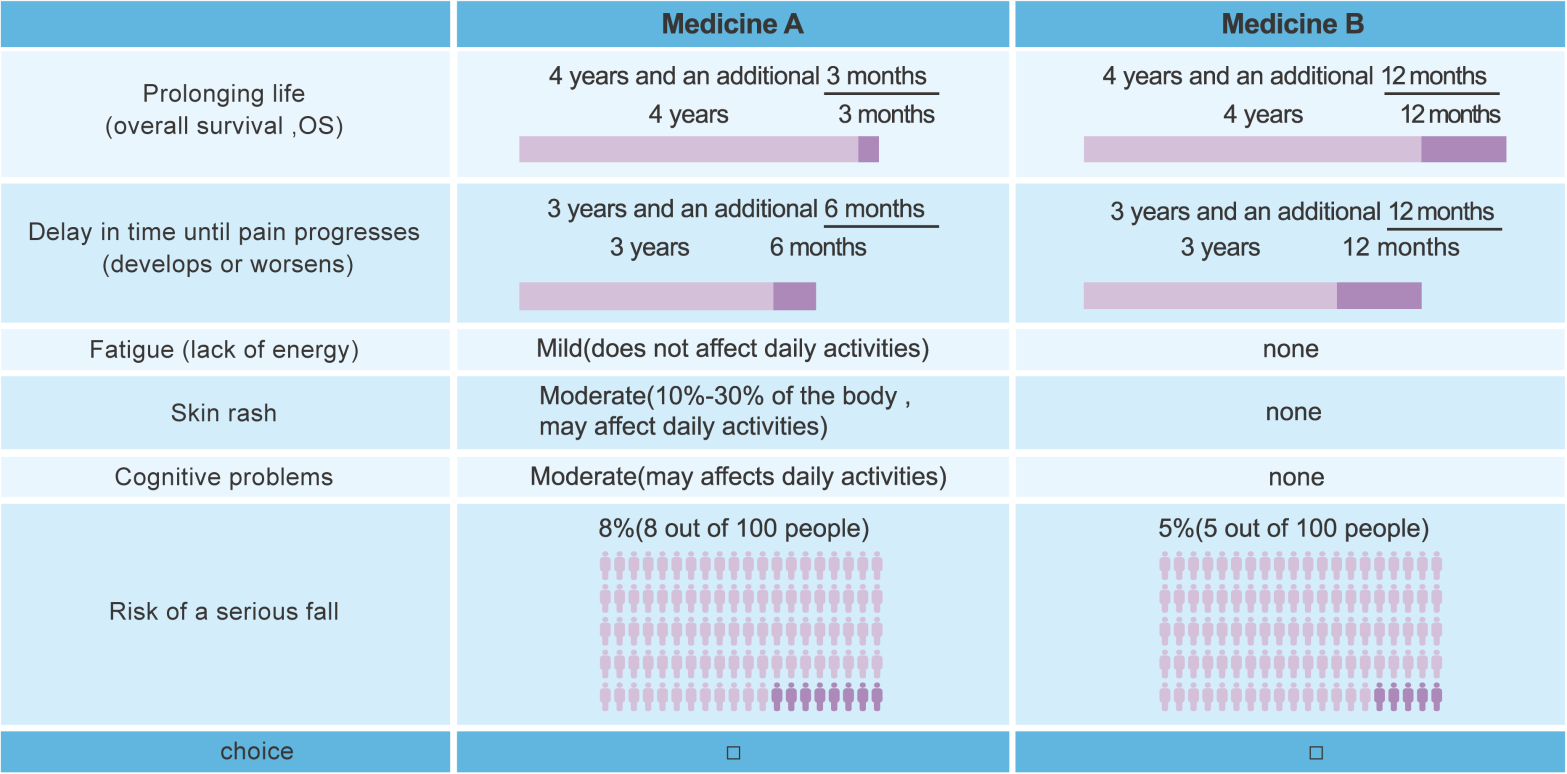
Example choice set.

The study began with a literature search and targeted population interviews to select 7 attributes: 2 efficacy attributes, including prolonged survival (OS) and time to pain progression (TPP; including onset or worsening), and 5 safety attributes, including fatigue, skin rash, cognitive problems, risk of serious fall, and risk of serious fracture. Serious fall is defined as the one result in injuries that require hospitalization, and serious fracture can be disabling, visually dislocated, and life-threatening. Besides, people taking the medicine may experience fatigue with felling of weak and lack of energy to do their daily activities, skin rash with symptoms like itchiness, burning sensation, and tightness, as well as cognitive problems such as confusion, memory loss, and inability to concentrate. Each attribute covered 3 different levels (see **Supplementary Table S1**). Applying a D-efficient fractional-factorial design, the attributes and levels were ranked and combined with a commonly used algorithm in SAS 9.4 software (SAS Institute) and a statistically valid set of preferred questions (22, 23). The final set consisted of 16 sets of DCE questionnaires with a total of 14 treatment choice questions per set, with each question comparing 2 hypothetical treatments that differed with regard to the risk or severity of 5 safety attributes (fatigue, skin rash, cognitive problems, serious fall, and serious fracture) and the duration of 2 efficacy attributes (OS and TPP). Questions answered by respondents were randomly selected from a predetermined pool of questions; there are 8 modules in the pool of questions (module 1–8), and each module consists of 7 questions. The medication for each question contains 6 attributes, while the sixth attribute of medications is different across modules, with the chance of a serious fall (attribute X6) in module 1–4 and the chance of a serious fracture (attribute X7) in module 5–8. An example is shown in **Figure 1**.

### 2.2 Participants

This is a paper-based DCE survey. We recruited physicians serving in urology or oncology department who had self-reported experience treating nmCRPC patients in their practice to complete the DCE questionnaire. Based on the DCE sample size calculation (24), 80 nmCRPC-treating physicians from urology or oncology departments were recruited for this study. The field work team recruited physicians through an offline contact model by asking physicians about their intention to participate in the study and asking them to complete screening questions. Eligibility was based on the following criteria: (1) ≥18 years of age, (2) licensed urologist or oncologist, and (3) with experience treating patients with nmCRPC. Physicians who had participated in a similar survey in the past 6 weeks or who were unwilling to provide informed consent were excluded. Physicians who met the inclusion and exclusion criteria were successfully entered into the study and completed a paper-based questionnaire.

To ensure the representativeness of the physician population included in this questionnaire study, the survey covered nmCRPC-treating urologists and oncologists from 7 administrative regions of mainland China, and quota sampling based on the geographic distribution of certified physicians from the China Health Statistical Yearbook 2021 was applied to ensure the geographic representativeness of the study population.

### 2.3 Procedures

Prior to the formal distribution of questionnaires, members of the study team conducted a face-to-face interview with 2 clinical experts who were asked to provide suggestions and comments on the feasibility and comprehensibility of the questionnaires. The study team then revised the questionnaires based on the feedback from the clinical experts, but the clinical experts’ data were not included in the final analysis. Members of the study team revised and finalized the final version of the DCE questionnaires and related sociodemographic and clinical experience questions based on the feedback from the clinical expert interviews. Before the main analysis was conducted, validity checks of the data were conducted. Physicians involved in situations of task nonattendance, attribute dominance, or monotonicity were excluded from the main analysis. In this study, each respondent was presented with 14 treatment choice questions in which they chose a preferred option between 2 hypothetical treatments (medication A and B). Since attributes X6 and X7 were not presented simultaneously, to eliminate the interference of the order of answers, a randomized grouping design was adopted, where physicians assigned with number 1 were first randomly allocated to one of module 1–4, then randomly allocated to one of module 5–8; whereas physicians assigned with number 2 were first randomly allocated to one of module 5–8, then randomly allocated to one of module 1–4.

### 2.4 Statistical analysis

The variables collected in this study include physicians’ sociodemographic characteristics, clinical experience, and the different attributes and levels included in the DCE model.

The sociodemographic characteristics and clinical experience of the study population were analyzed using descriptive statistics. For continuous variables, the mean and standard deviation were calculated; for categorical variables, the frequencies and corresponding percentages were calculated.

DCE responses were analyzed using the random parameters logit (RPL) model to produce the preference weight for each attribute level (25). The RPL model generates preference weights for each attribute level, with larger weights indicating stronger preferences. The relative attribute importance score (RAIS) was also implemented to describe the relative influence of each attribute on physicians’ treatment choices. Moreover, the marginal rate of substitution (MRS) was used to quantify the amount of OS physicians were willing to trade in return for a reduction in AEs. All DCE analyses used were performed using Stata IC 14.2 (StataCorp) and R Studio 3.5.0.

This study was conducted as an exploratory analysis that stratified physicians according to their sociodemographic characteristics and clinical experience to examine whether there were differences between subgroups in the RAIS of treatment benefit–risk considerations and MRS.

## 3 Results

### 3.1 Basic characteristics

A total of 80 nmCRPC-treating physicians completed the survey. No physician was excluded from the main analysis due to violation of validity checks. The sociodemographic characteristics of the 80 physicians are shown in **Table 1**, and information of their clinical experience in nmCRPC management is available in **Supplementary Table S2**. The average time in clinical practice for all participating physicians was about 18.4 years.

**Table 1:** Sociodemographic characteristics of the 80 physicians.

Characteristic	Value
**Age, years**	45 [7.13]
Gender, n (%)	
Male	62 (77.5)
Female	18 (22.5)
Hospital location	
North China	12 (15.0)
East China	25 (31.3)
Northeast China	6 (7.5)
Central China	12 (15.0)
South China	9 (11.3)
Southwest China	11 (13.8)
Northwest China	5 (6.3)
** Duration of clinical practice, years**	18.4 [7.39]
Department	
Urology	51 (63.8)
Medical oncology	28 (35.0)
Surgical oncology	1 (1.3)
Title	
Attending physician	20 (25.0)
Associate chief physician	42 (52.5)
Chief physician	18 (22.5)

Data were presented as N (%) or mean [standard deviation].

### 3.2 Physician preferences and relative importance of attributes

**Figure 2:**
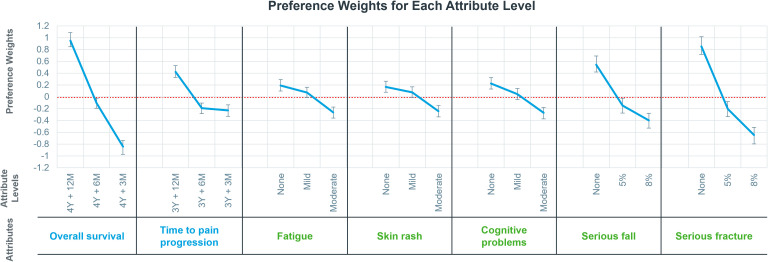
Preference weights for each attribute level. Preference weights indicate physician preferences of each attribute level, signs of preference weights indicate physicians’ positive or negative attitudes, and the absolute values of preference weights represent the preference level, with the larger the absolute value, the stronger the physicians’ preference. A preference weight close to zero indicates that physicians have no preference on this level. The horizontal coordinate is the 7 attributes and the 3 levels of the corresponding attributes. Y, year(s); M, month(s).

In general, physician preferences were logically ordered, with higher efficacy and lower risks for AEs being favored over lower efficacy and higher risks for AEs (**Figure 2**). **Figure 2** shows the preference weights for each attribute level. Physicians expressed strong preferences toward OS, risk of serious fracture, risk of serious fall, and TPP. For the second level of fatigue, skin rash, and cognitive problems, physicians’ preferences were not significantly different from zero, which suggests that physicians felt indifferent toward the level of these 3 attributes.

**Figure 3:**
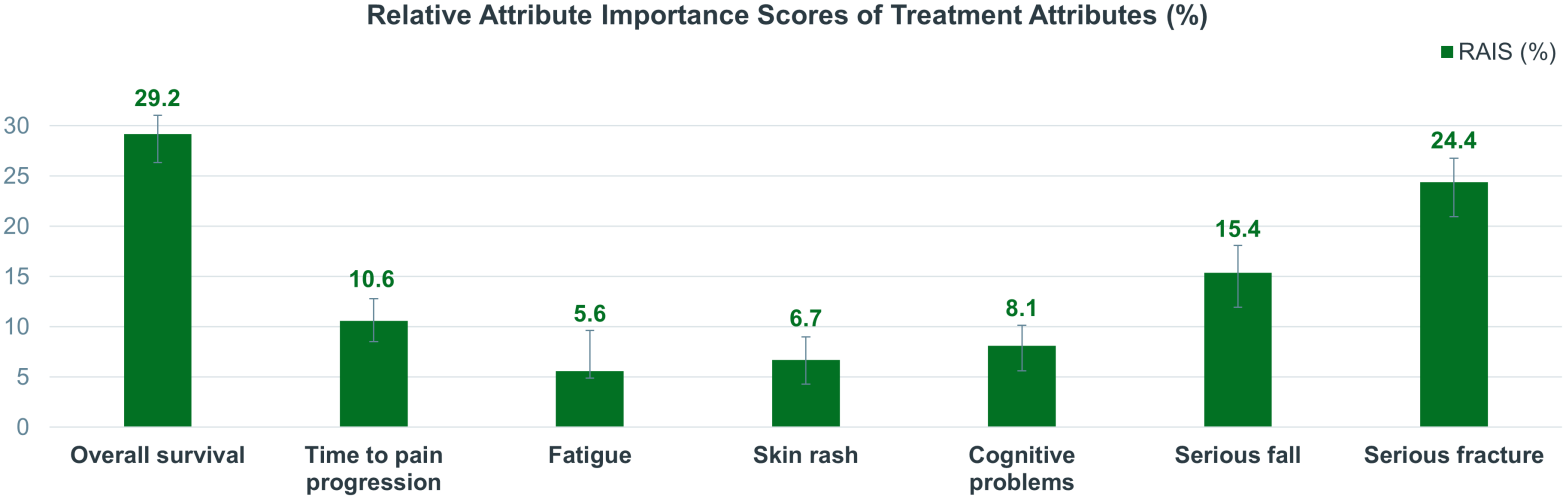
Relative attribute importance scores of treatment attributes (%). The larger the RAIS of an attribute, the greater the impact physicians perceived with a change on the attribute’s level. RAIS, relative attributes importance score.

Regarding the relative importance of various attributes, physicians focused more on OS in terms of efficacy and on serious fracture in terms of safety. With regard to efficacy attributes, physicians placed greater emphasis on OS than on TPP. In terms of safety attributes, serious fracture was perceived as the most important AE by physicians, followed by serious fall, cognitive problems, skin rash, and fatigue (**Figure 3**). Furthermore, the RAIS demonstrated that the importance of severe fracture was second only to the importance of OS.

### 3.3 Tradeoffs between OS and AEs

**Figure 4:**
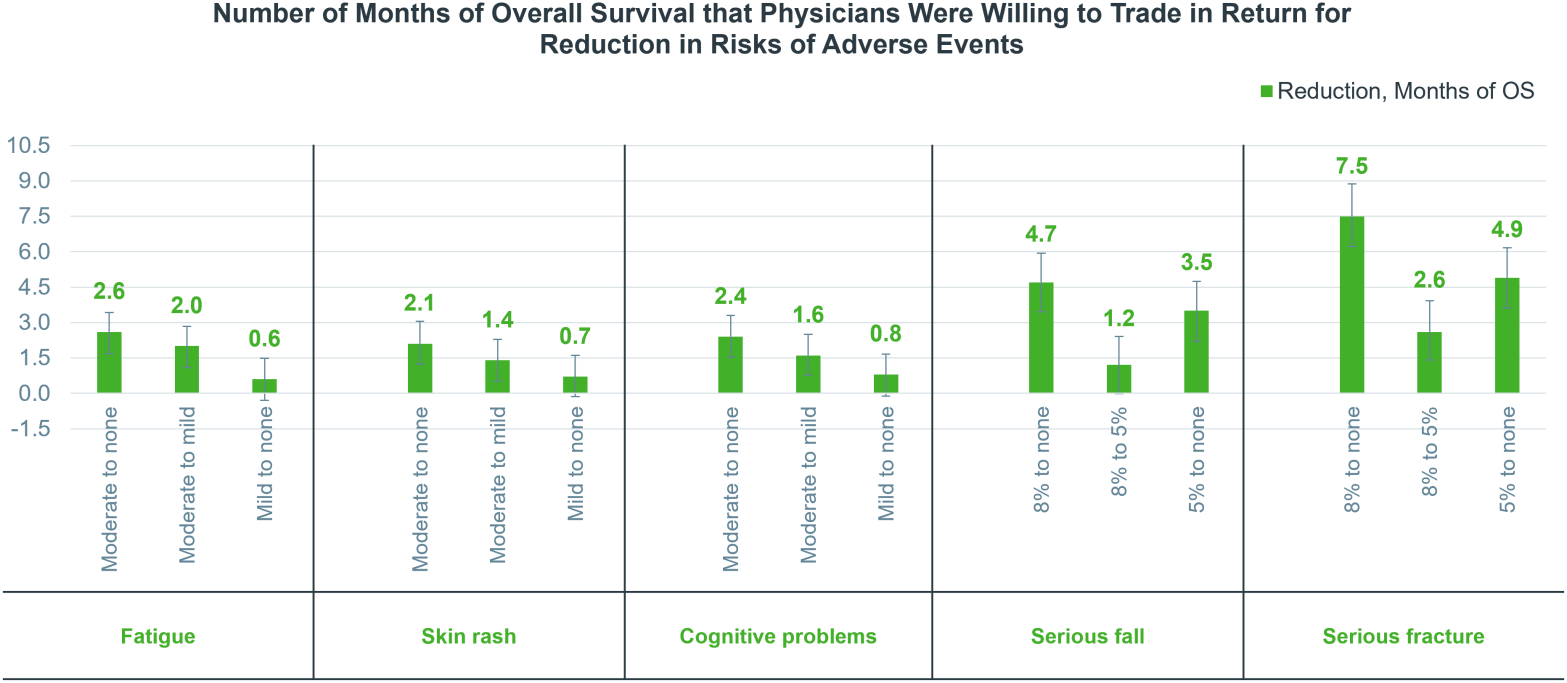
Number of months of overall survival that physicians were willing to trade in return for a reduction in risks of adverse events. OS, overall survival.

**Figure 4** presents the amount of OS that physicians were willing to forego for a reduction in AEs. In general, physicians were willing to trade more OS to reduce the risk of serious fracture compared to other safety attributes. For serious fracture and serious fall, physicians were on average willing to forego 7.5 and 4.7 months of OS, respectively, in return for a reduction in the risk of serious fracture and serious fall from 8% to none. Moreover, a substantial reduction from 8% to none was more preferred by physicians for both attributes.

In addition, among fatigue, skin rash, and cognitive problems, physicians emphasized the risk reduction of fatigue the most. Specifically, to reduce the severity of fatigue, cognitive problems, and skin rash from moderate to none, physicians were willing to trade 2.6, 2.4, and 2.1 months of OS, respectively, and a substantial reduction from moderate to no risk was more attractive to physicians.

### 3.4 Subgroup analysis

Exploratory analysis was conducted by stratifying physicians into subgroups (see **Table 2**) and by examining the RAIS physicians placed on treatment benefits versus risks and the OS tradeoff in each subgroup (**Supplementary Figure S1**).

**Table 2:** Subgroup characteristics of the 80 physicians.

Subgroup characteristic	N (%)
Duration of clinical practice, years	
≤15	32 (40.0)
>15	48 (60.0)
Department	
Urology	51 (63.8)
Oncology	29 (36.3)
Title	
Attending Physician and below	20 (25.0)
Associate Chief Physician and above	60 (75.0)
Number of patients with nmCRPC treated in the past year	
≤ 20	43 (53.8)
> 20	37 (46.3)
Hospital location	
North China, East China, and South China	46 (57.5)
Central China, Northeast China, Southwest China, and Northwest China	34 (42.5)

nmCRPC, nonmetastatic castration-resistant prostate cancer.

#### 3.4.1 Duration of clinical practices

In a comparison of attributes among different groups, physicians with longer duration of clinical practice placed more emphasis on OS, risk of serious fracture, and risk of serious fall and were willing to trade more OS in return for a reduction in the risk of fatigue, skin rash, and cognitive problems; meanwhile, physicians with less experience showed greater concern for TTP, skin rash, and cognitive problems.

#### 3.4.2 Department

Urologists were concerned more about serious fracture, TPP, serious fall, and skin rash, and oncologists placed more emphasis on OS, cognitive problems, and fatigue. As compared to oncologists, urologists in general were willing to forego more OS to reduce the risk of AEs except for cognitive problems.

#### 3.4.3 Title

When weighing benefits and risks, associate chief physicians and those in positions above this level placed more emphasis on OS, serious fracture, and serious fall, with the other efficacy and safety attributes being perceived as more important among attending physicians and those in positions below this level. Generally, attending physicians and below were willing to forego more OS to reduce AEs, except for the reduction of risk of serious fall from 8% to 5% or none and serious fracture from 5% to none.

#### 3.4.4 Number of patients with nmCRPC treated in the past year

Physicians who had treated more than 20 patients with nmCRPC in the past year tended to place more value on OS and were less willing to forego OS for AEs, while physicians treating fewer patients focused more on TPP, skin rash, cognitive problems, and serious fall.

#### 3.4.5 Hospital location

Physicians from regions with more developed economies, including North, East, and South China, tended to place more value on the reduction of AEs and were willing to trade more OS in return for a reduction in risks of AEs, especially for serious fracture and serious fall. In particular, physicians from regions with less developed economies, including Central, Northeast, Southwest, and Northwest China, placed the most importance on OS among all attributes.

## 4 Discussion

Our study used the DCE approach to analyze the relative importance of various attributes of nmCRPC physicians and the extent to which they were willing to forego treatment-related benefits (i.e., several months of OS). Our findings showed that although physicians attached the greatest importance to OS, they also thought highly of avoiding AEs, and among all safety attributes, physicians were most concerned about serious fracture, with an RAIS only second to OS, followed by serious fall, cognitive impairment, rash, and fatigue. The physicians’ emphasis on the risk of serious fracture and serious fall may be due to the fact that patients with nmCRPC are mostly older males with multiple comorbidities, and severe fracture and fall may affect their quality of life or even cause deformity and death (26). In addition, physicians need to pay more attention to cognitive impairment in patients with nmCRPC which may be present due to ADT treatment (27, 28). Considering the relative importance of physicians’ different treatment safety attributes and how much OS they will forego to reduce the risk of AEs together with patients’ potential risk of developing some specific AEs, we can help physicians address an important gap in the development of personalized treatment plans for nmCRPC patients.

Similar DCE studies on nmCRPC treatment preferences of physicians have been conducted in the United States and Japan. The study by Srinivas et al. showed that when weighing both benefits and risks, different from Chinese physicians, US physicians were most concerned with AEs, and they placed most emphasis on cognitive problems, followed by serious fracture, fatigue, serious falls, and rash, while they ranked the relative importance of OS only third among all attributes. On the contrary, Chinese physicians expressed strong preferences toward OS, serious fracture, serious fall, and TPP, with cognitive problems only ranking fifth. Moreover, US physicians were willing to trade 11.6 and 9.2 months of OS, respectively, to reduce the risk of cognitive problems and serious fracture from severe to none, while Chinese physicians were on average willing to forego 7.5 and 4.7 months of OS, respectively, in return for a reduction in the risk of serious fracture and serious fall from 8% to none, and only 2.4 months of OS to reduce the risk of cognitive problems from moderate to none (13). There is still a significant difference between Chinese and US physicians when weighing treatment benefits and risk preferences. And US physicians placed more emphasis on treatment-related risks, especially cognitive problems, whereas Chinese physicians attach more importance to treatment-related benefits. In Japan, the results of Suzuki et al. showed that similar to Chinese physicians, Japanese physicians also placed the most emphasis on treatment benefits compared to AEs, and among safety attributes, Japanese physicians were likely most concerned with the risk of serious fall or fracture and risk of fatigue. The risk of cognitive impairment, risk of hypertension, and risk of rashes were deemed less important compared with the other safety attributes (29). Thus, we can find a more consistent treatment preference among Asian physicians. However, it still remains to be seen whether these treatment preferences hold in the real world.

Analysis of nmCRPC patients’ and caregivers’ preferences for treatment choices is also essential. We sought to characterize patient and caregiver perspectives in mainland China. Patients with nmCRPC were most concerned with serious fracture, followed by serious fall and cognitive problems, which could severely affect patients’ HRQoL. In contrast, caregivers placed more importance on serious fracture, cognitive problems, and OS, which could increase the difficulty of caregiving. In addition, patients and caregivers were willing to trade 15.9 and 11.0 months, 12.5 and 9.2 months, and 11.2 and 9.9 months, respectively, for a reduction in the risk of serious fracture, serious fall, and cognitive problems from 8% or moderate to none (30). This shows that patients and caregivers placed greater weight on risks of serious fracture, serious fall, and cognitive problems and were even willing to forego a certain amount of survival time for a reduction in these AEs. Meanwhile, our study found that physicians also placed a high value on these attributes. Therefore, physicians and patients from mainland China are relatively consistent regarding the attributes they focus on although there were differences in the ranking of the relative importance of each attribute, with physicians focusing most on OS and patients focusing most on the risk of serious fracture, with OS ranking only fourth. This is also reflected in the choice of survival time in exchange for the reduced risk of AEs, where patients were prepared to give up more survival time. Therefore, when physicians consider how to prolong patient survival time, they should not ignore patients’ demands for quality of life.

Another important aspect of this study lay in the exploratory analysis of stratifying physicians according to their sociodemographic characteristics and clinical experience and in exploring the tradeoff between benefits and risks in each subgroup. First, physicians with longer duration of clinical practice, associate chief physicians and above, and those who had treated more patients with nmCRPC in the past year placed more emphasis on prolonged OS and were more reluctant to forego patient survival to reduce the risk of most AEs. In contrast, physicians with less clinical practice, attending physicians and below, and those who had treated fewer patients with nmCRPC in the past year were more concerned about AEs and were willing to give up more of the patients’ OS to reduce the risk of such events. This suggests that physicians’ practice duration and experience may influence the tradeoff between treatment benefits and AEs. Second, both urologists and oncologists placed the most importance on prolonged survival and serious fracture, and urologists were willing to give up more of their patients’ survival time to reduce the risk of most AEs. We also found that physicians from more economically developed regions placed more emphasis on reducing the risk of AEs and were willing to forego more OS. Thus, we can speculate that physicians in more economically developed areas are more focused on the life quality of patients.

We recognize that this study also has some limitations. The main limitation of using the DCE approach is the need to make judgments about hypothetical choices, which may result in hypothesis bias. Given the unavailability of real-world data on physician prescriptions, a controlled study in the form of DCE serves as the best data source. Our options were obtained from feedback from qualitative interviews, and hypothesis bias was reduced by constructing options that simulated realistic clinical choices. Additionally, the sample size of most subgroups in the subgroup analysis did not meet the minimum sample size (n=54), and there was no design or mandate to detect statistical differences in the subgroups. Thus, there may be some bias in the results of the analysis across subgroups, and these differences should be interpreted with caution.

## 5 Conclusions

Physicians from mainland China valued the importance of minimizing treatment-related AEs when considering different treatment options for patients with nmCRPC. With regard to safety attributes, serious fracture was perceived as the most important AE by physicians, followed by serious fall, cognitive problems, skin rash, and fatigue. Moreover, they were willing to forego a substantial amount of OS to avoid AEs between hypothetical treatments. The results demonstrated that the quality of life is critical for patients with nmCRPC, and in devising therapeutic regimens, it is essential to balance the treatment-related benefits and risks between novel SGARI therapies.

## Data availability statement

The original contributions presented in the study are included in the article/**Supplementary Material**. Further inquiries can be directed to the corresponding author.

## Ethics statement

The studies involving humans were approved by The Biomedical Research Ethics Committee of Peking University First Hospital. The studies were conducted in accordance with the local legislation and institutional requirements. The participants provided their written informed consent to participate in this study.

## Author contributions

YF: Data curation, Formal analysis, Investigation, Methodology, Writing – original draft. XG: Data curation, Formal analysis, Investigation, Methodology, Writing – original draft. DC: Writing – review & editing. ZH: Conceptualization, Supervision, Writing – review & editing.

## References

[1] Lokeshwar SD Klaassen Z Saad F. Treatment and trials in non-metastatic castration-resistant prostate cancer. Nat Rev Urol. (2021) 18:433–42. doi: 10.1038/s41585-021-00470-4 34002069

[2] Hird AE Dvorani E Saskin R Emmenegger U Herschorn S Kodama R. Prevalence and natural history of non-metastatic castrate resistant prostate cancer: A population-based analysis. Clin Genitourin Cancer. (2023) 21:e27–34. doi: 10.1016/j.clgc.2022.10.003 36371403

[3] Kirby M Hirst C Crawford ED. Characterising the castration-resistant prostate cancer population: a systematic review. Int J Clin Pract. (2011) 65:1180–92. doi: 10.1111/ijcp.2011.65.issue-11 21995694

[4] de Sá Moreira E Robinson D Hawthorne S Zhao L Hanson M Kanas G. Patterns of care and outcomes for non-metastatic prostate cancer in the United States: results of the cancerMPact(®) survey 2018. Cancer Manag Res. (2021) 13:9127–37. doi: 10.2147/CMAR.S343321 PMC8674664 34924773

[5] Tombal B. Non-metastatic CRPC and asymptomatic metastatic CRPC: which treatment for which patient? Ann Oncol. (2012) 23 Suppl 10:x251–8. doi: 10.1093/annonc/mds325 22987972

[6] Fizazi K Shore N Tammela TL Ulys A Vjaters E Polyakov S. Nonmetastatic, castration-resistant prostate cancer and survival with darolutamide. N Engl J Med. (2020) 383:1040–9. doi: 10.1056/NEJMoa2001342 32905676

[7] Gupta R Sheng IY Barata PC Garcia JA. Non-metastatic castration-resistant prostate cancer: current status and future directions. Expert Rev Anticancer Ther. (2020) 20:513–22. doi: 10.1080/14737140.2020.1772759 32508166

[8] López-Campos F Conde-Moreno A Barrado Los Arcos M Gómez-Caamaño A García-Gómez R Hervás Morón A. Treatment landscape of nonmetastatic castration-resistant prostate cancer: A window of opportunity. J Pers Med. (2021) 11:1190. doi: 10.3390/jpm11111190 34834544 PMC8619952

[9] Kelly R Anton A Wong S Shapiro J Weickhardt A Azad A. Real-world use of first-generation antiandrogens: impact on patient outcomes and subsequent therapies in metastatic castration-resistant prostate cancer. BJU Int. (2021) 128 Suppl;1:18–26. doi: 10.1111/bju.15364 34622543

[10] Tartarone A Lerose R Tartarone M. Decisions and dilemmas in non-metastatic castration-resistant prostate cancer management. Med Oncol. (2022) 39:107. doi: 10.1007/s12032-022-01743-7 35553247

[11] Saad F Bögemann M Suzuki K Shore N. Treatment of nonmetastatic castration-resistant prostate cancer: focus on second-generation androgen receptor inhibitors. Prostate Cancer Prostatic Dis. (2021) 24:323–34. doi: 10.1038/s41391-020-00310-3 PMC8134049 33558665

[12] Hussain M Fizazi K Saad F Rathenborg P Shore N Ferreira U. Enzalutamide in men with nonmetastatic, castration-resistant prostate cancer. N Engl J Med. (2018) 378:2465–74. doi: 10.1056/NEJMoa1800536 PMC8288034 29949494

[13] Srinivas S Mohamed AF Appukkuttan S Botteman M Ng X Joshi N. Physician preferences for non-metastatic castration-resistant prostate cancer treatment. BMC Urol. (2020) 20:73. doi: 10.1186/s12894-020-00631-4 32571276 PMC7310549

[14] Brave M Weinstock C Brewer JR Chi DC Suzman DL Cheng J. An FDA review of drug development in nonmetastatic castration-resistant prostate cancer. Clin Cancer Res. (2020) 26:4717–22. doi: 10.1158/1078-0432.CCR-19-3835 32284318

[15] Swami U Agarwal N. Improvement in overall survival with Apalutamide, Darolutamide and Enzalutamide in patients with non-metastatic castration-resistant prostate cancer. Cancer Treat Res Commun. (2020) 25:100205. doi: 10.1016/j.ctarc.2020.100205 32822968

[16] Feng FY Thomas S Saad F Gormley M Yu MK Ricci DS. Association of molecular subtypes with differential outcome to apalutamide treatment in nonmetastatic castration-resistant prostate cancer. JAMA Oncol. (2021) 7:1005–14. doi: 10.1001/jamaoncol.2021.1463 PMC8176389 34081076

[17] Smith MR Saad F Chowdhury S Oudard S Hadaschik BA Graff JN. Apalutamide treatment and metastasis-free survival in prostate cancer. N Engl J Med. (2018) 378:1408–18. doi: 10.1056/NEJMoa1715546 29420164

[18] Hussain M Fizazi K Saad F Rathenborg P Shore ND Demirhan E. PROSPER: A phase 3, randomized, double-blind, placebo (PBO)-controlled study of enzalutamide (ENZA) in men with nonmetastatic castration-resistant prostate cancer (M0 CRPC). J Clin Oncol. (2018) 36:3. doi: 10.1200/JCO.2018.36.6_suppl.3

[19] Smith MR Hussain M Saad F Fizazi K Sternberg CN Crawford ED. Darolutamide and survival in metastatic, hormone-sensitive prostate cancer. N Engl J Med. (2022) 386:1132–42. doi: 10.1056/NEJMoa2119115 PMC9844551 35179323

[20] Halabi S Jiang S Terasawa E Garcia-Horton V Ayyagari R Waldeck AR. Indirect comparison of darolutamide versus apalutamide and enzalutamide for nonmetastatic castration-resistant prostate cancer. J Urol. (2021) 206:298–307. doi: 10.1097/JU.0000000000001767 33818140

[21] Shore ND Stenzl A Pieczonka C Klaassen Z Aronson WJ Karsh L. Impact of darolutamide on local symptoms: pre-planned and *post hoc* analyses of the ARAMIS trial. BJU Int. (2023) 131:452–60. doi: 10.1111/bju.15887 36087070

[22] Reed Johnson F Lancsar E Marshall D Kilambi V Mühlbacher A Regier DA. Constructing experimental designs for discrete-choice experiments: report of the ISPOR Conjoint Analysis Experimental Design Good Research Practices Task Force. Value Health. (2013) 16:3–13. doi: 10.1016/j.jval.2012.08.2223 23337210

[23] Kuhfeld WF. Marketing research methods in SAS: experimental design, choice, conjoint, and graphical techniques. Cary, NC: SAS Institute Inc (2010). SAS 9.2 Edition.

[24] de Bekker-Grob EW Donkers B Jonker MF Stolk EA. Sample size requirements for discrete-choice experiments in healthcare: a practical guide. Patient. (2015) 8:373–84. doi: 10.1007/s40271-015-0118-z PMC4575371 25726010

[25] Hauber AB González JM Groothuis-Oudshoorn CG Prior T Marshall DA Cunningham C. Statistical methods for the analysis of discrete choice experiments: A report of the ISPOR conjoint analysis good research practices task force. Value Health. (2016) 19:300–15. doi: 10.1016/j.jval.2016.04.004 27325321

[26] Myint ZW Momo HD Otto DE Yan D Wang P Kolesar JM. Evaluation of fall and fracture risk among men with prostate cancer treated with androgen receptor inhibitors: A systematic review and meta-analysis. JAMA Netw Open. (2020) 3:e2025826. doi: 10.1001/jamanetworkopen.2020.25826 33201234 PMC7672516

[27] Mateo J Fizazi K Gillessen S Heidenreich A Perez-Lopez R Oyen WJG. Managing nonmetastatic castration-resistant prostate cancer. Eur Urol. (2019) 75:285–93. doi: 10.1016/j.eururo.2018.07.035 30119985

[28] Ryan C Wefel JS Morgans AK. A review of prostate cancer treatment impact on the CNS and cognitive function. Prostate Cancer Prostatic Dis. (2020) 23:207–19. doi: 10.1038/s41391-019-0195-5 PMC7237350 31844181

[29] Suzuki K Grillo V Chen Y Singh S Ledesma DA. Understanding treatment strategies and preferences in nonmetastatic castration-resistant prostate cancer from the Japanese physician perspective. JCO Glob Oncol. (2021) 7:302–10. doi: 10.1200/GO.20.00358 PMC8081502 33617305

[30] Jindan L. Preferences for non-metastatic castration-resistant prostate cancer treatments: A discrete choice experiment among patient and caregiver in China. Available online at: https://www.siu-urology.org/flipbooks/2022_abstract_book/html5forwebkit.html.

