# Supplementary material for: Physician preferences for nonmetastatic castration-resistant prostate cancer treatment in China
Source: Front Oncol. 2024 May 21;14:1382678. doi: 10.3389/fonc.2024.1382678 (PMC11148332; doi:10.3389/fonc.2024.1382678)
Supplement: Supplementary file 1 [file DataSheet_1.docx]

**Physician preferences for nonmetastatic castration-resistant prostate cancer treatment in China**

**Supplementary Appendix**

**Appendix 1**

**Figure S1 RAIS and OS tradeoff (reduction in months of OS) by subgroup analysis (A-J).** (A) RAIS by duration of clinical practice. (B) OS tradeoff (reduction in months of OS) by duration of clinical practice. (C) RAIS by department of physicians. (D) OS tradeoff (reduction in months of OS) by department of physicians. (E) RAIS by title of physicians. (F) OS tradeoff (reduction in months of OS) by title of physicians. (G) RAIS by number of patients with nmCRPC treated in the past year. (H) OS tradeoff (reduction in months of OS) by number of patients with nmCRPC treated in the past year. (I) RAIS by hospital location. (J) OS tradeoff (reduction in months of OS) by hospital location. RAIS = relative attributes importance score; OS = overall survival. Subgroup analysis results are only for reference. Both subgroups did not exceed the minimum sample size requirement of 54.

**eFigure 1 (A)**


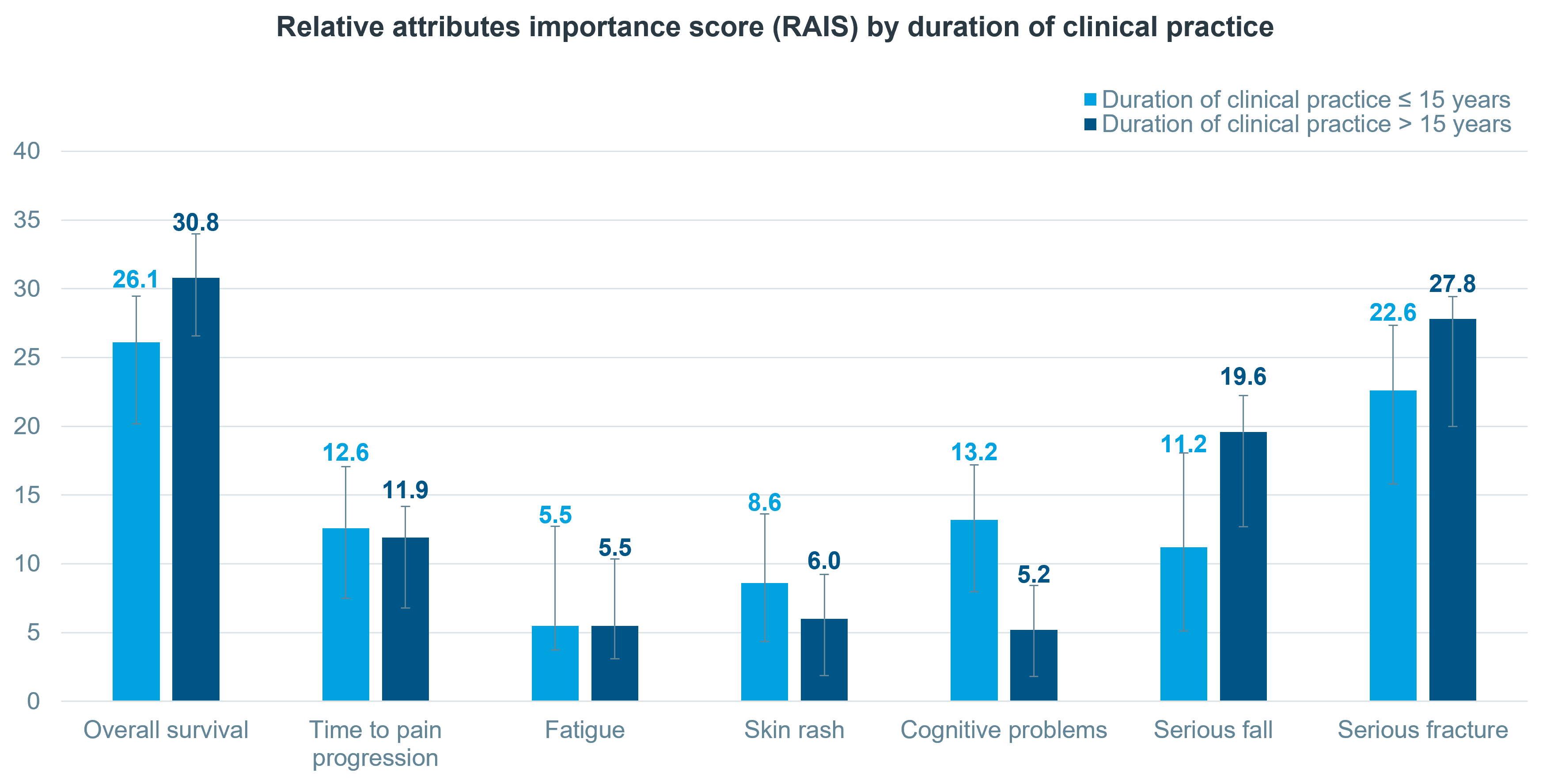


**eFigure 1 (B)**


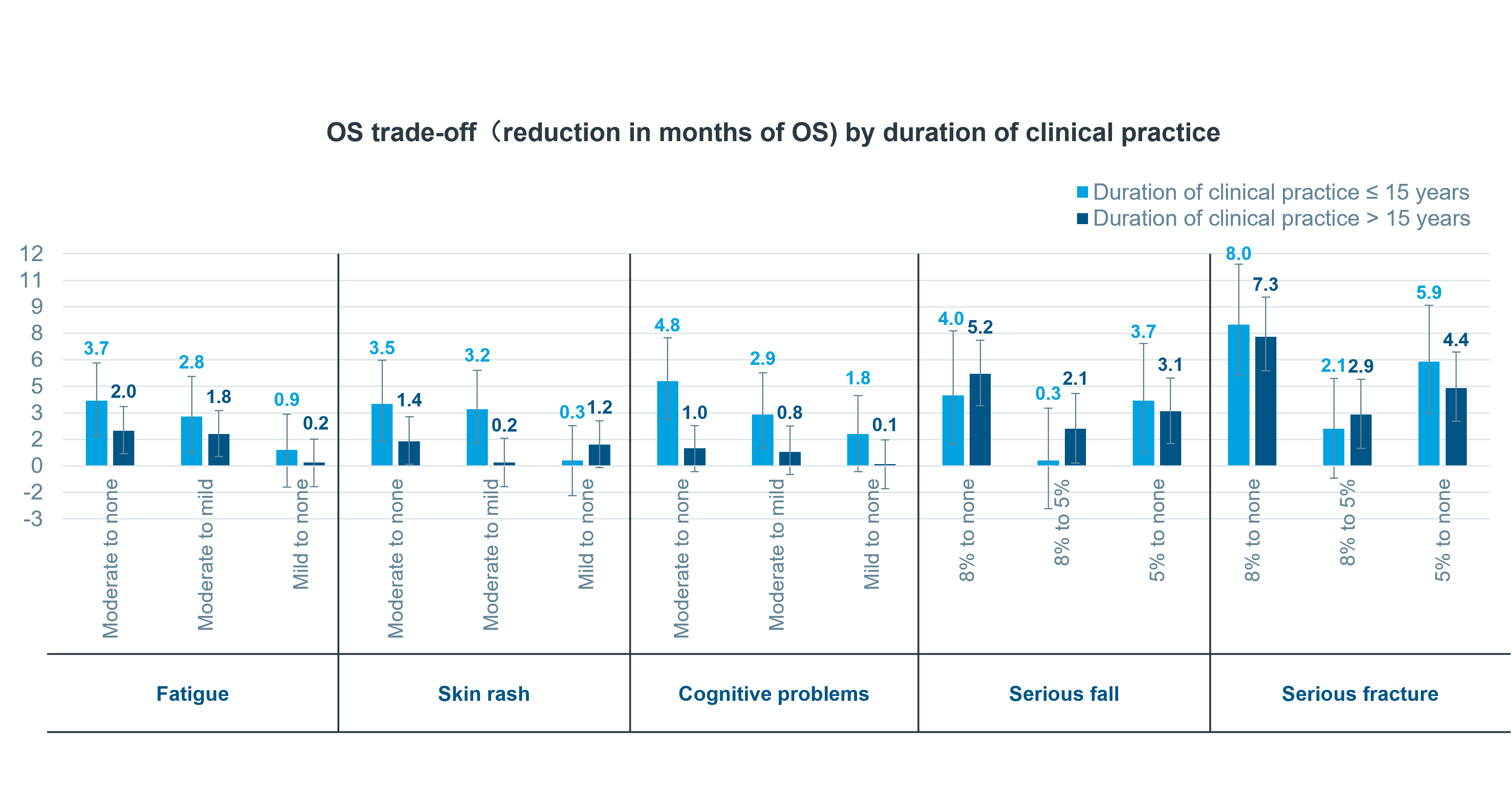


**eFigure 1 (C)**


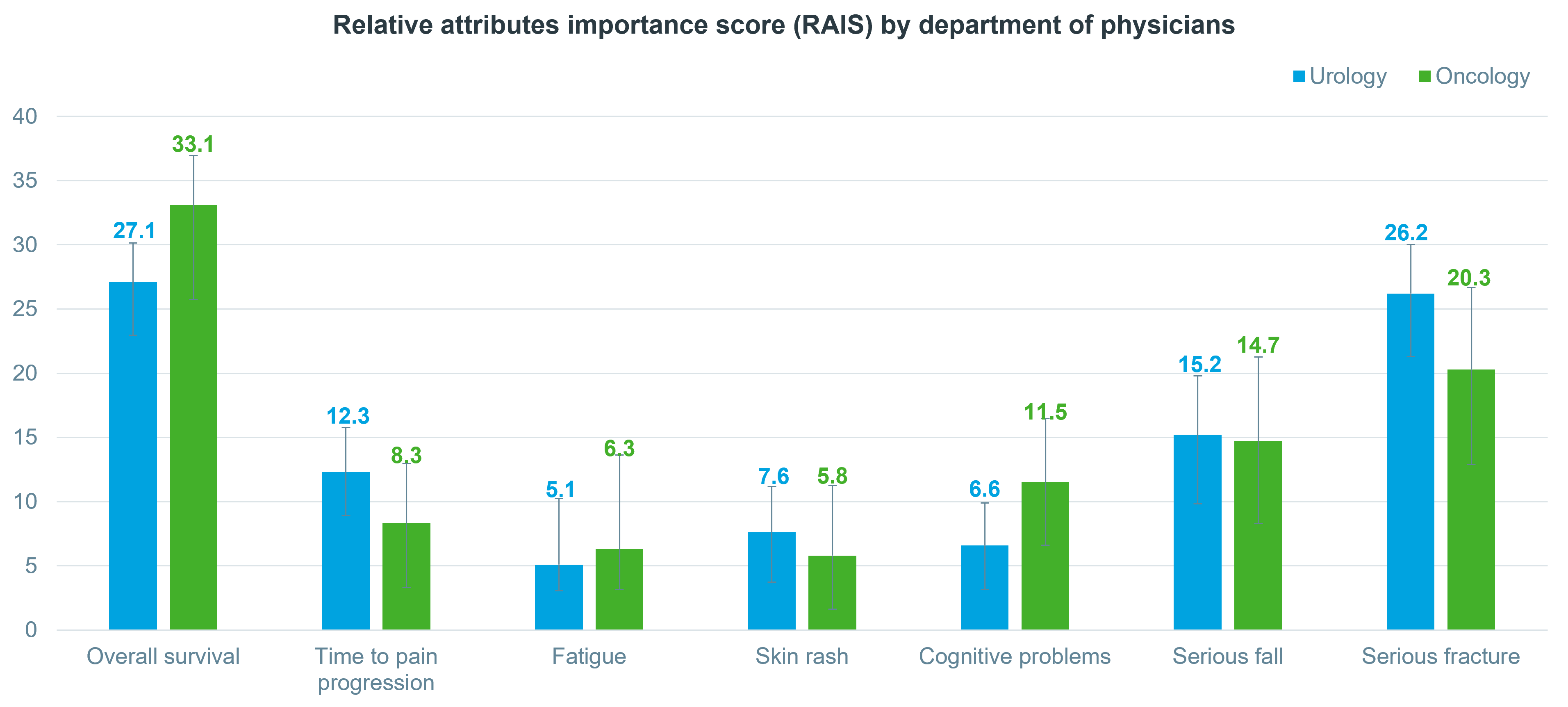


**eFigure 1 (D)**

**
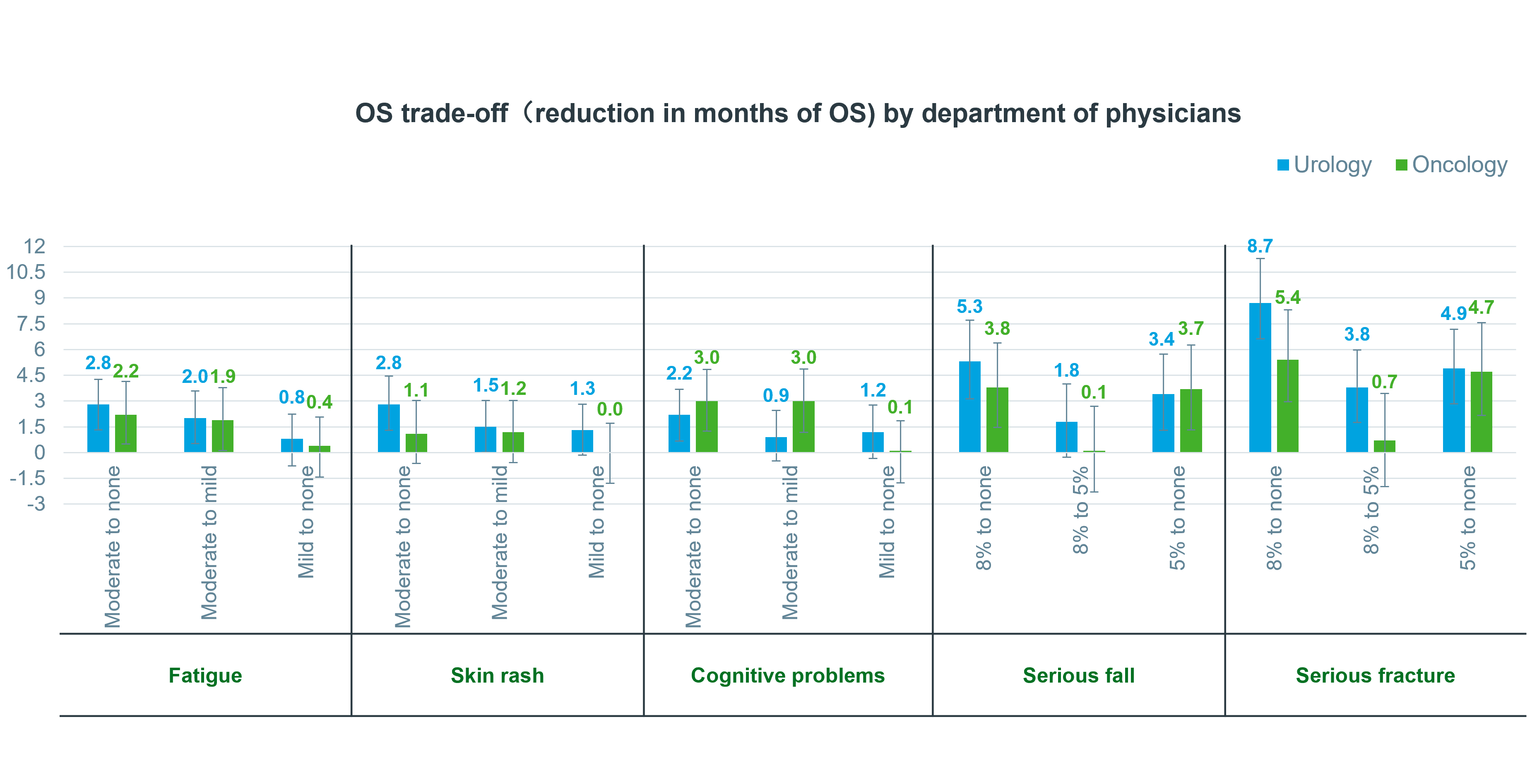
**

**eFigure 1 (E)**

**
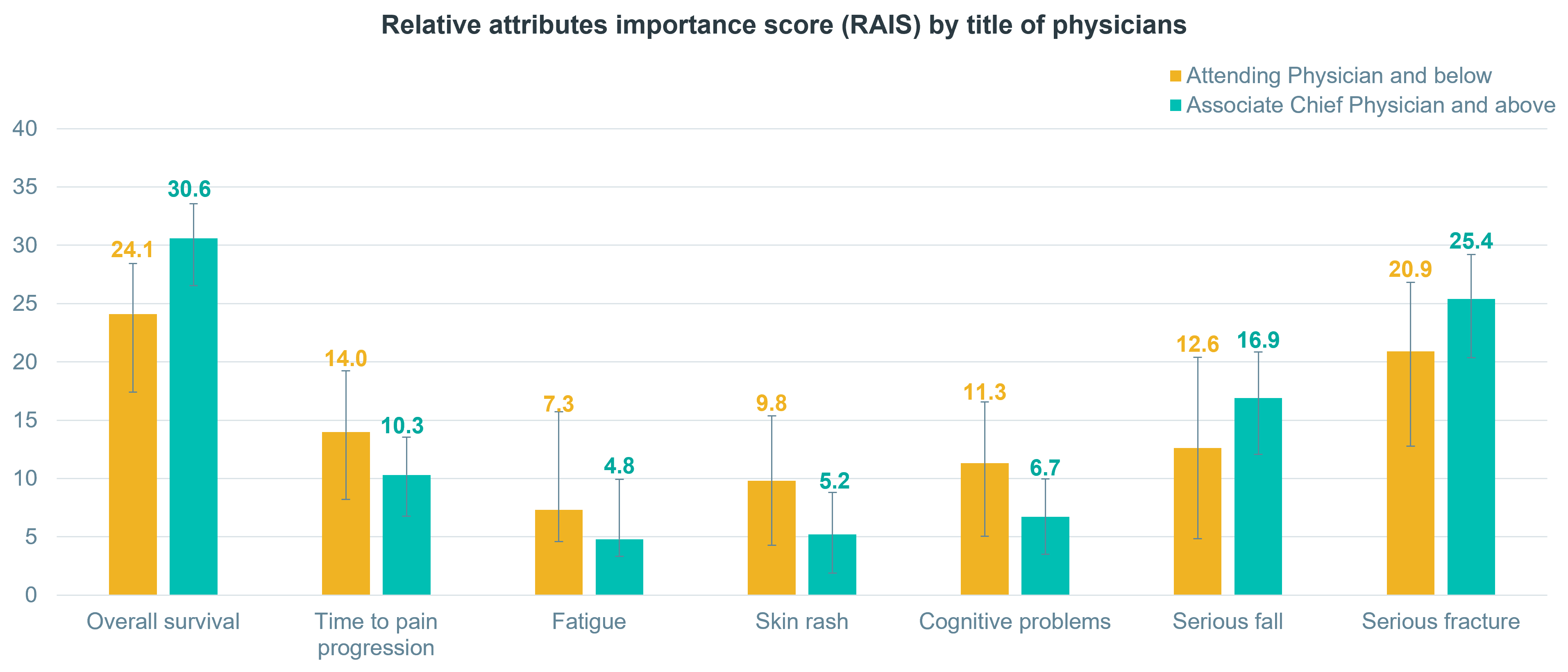
**

**eFigure 1 (F)**


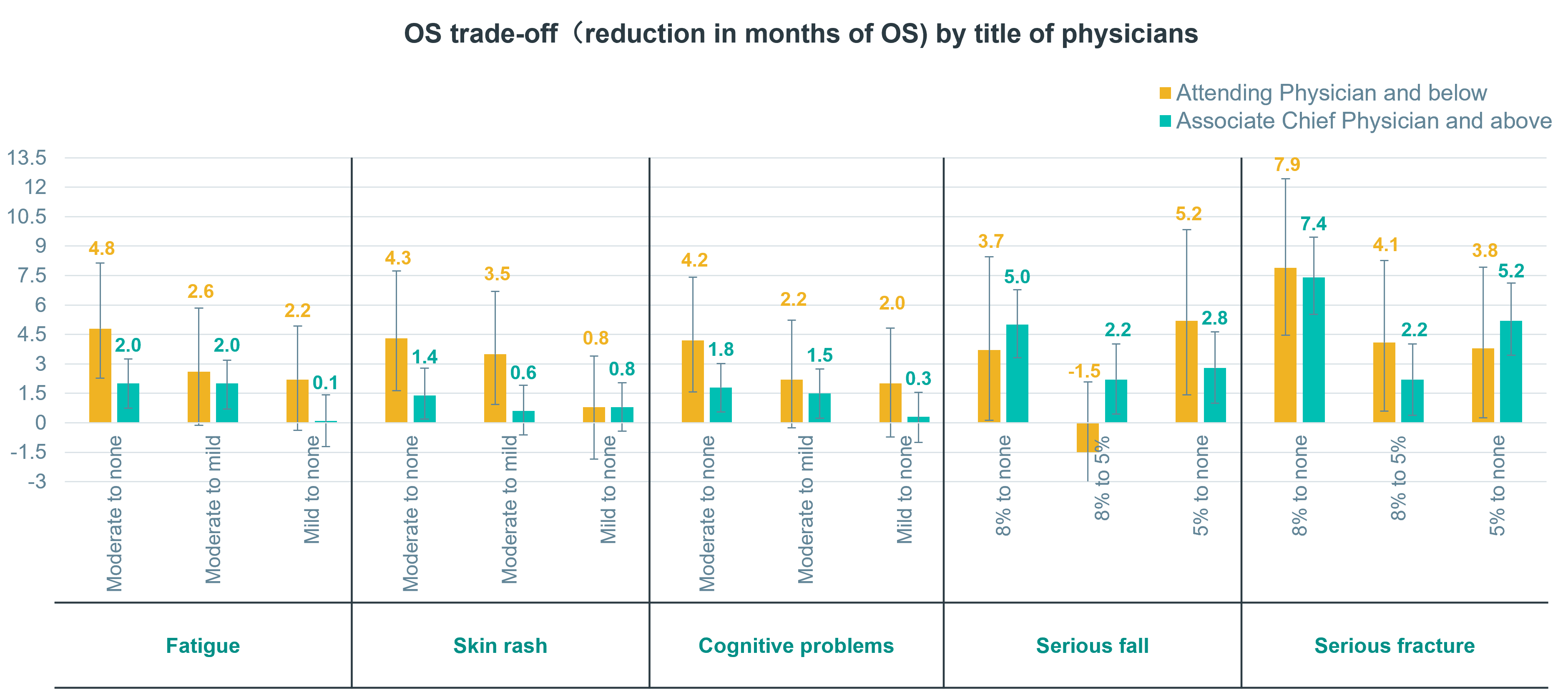


**eFigure 1 (G)**

**
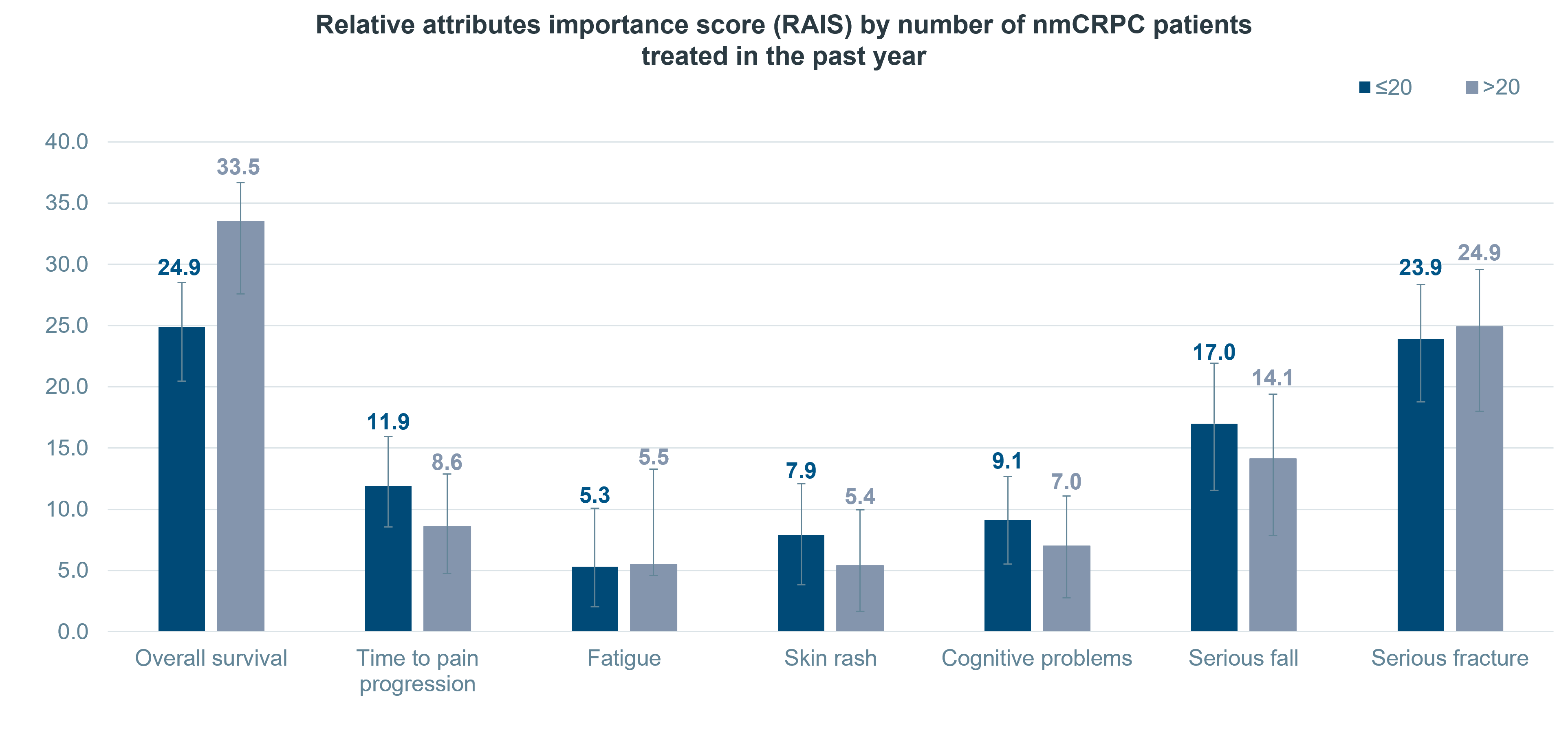
**

**eFigure 1 (H)**


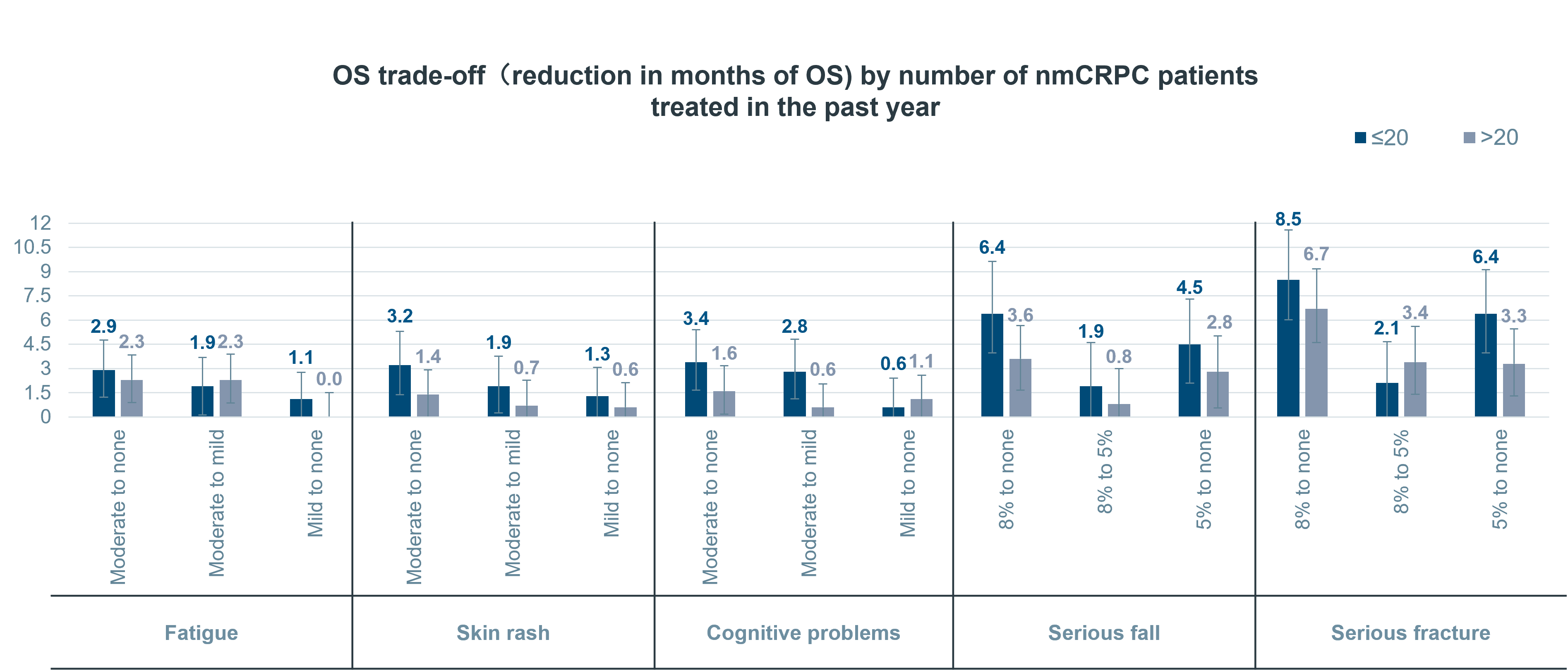


**eFigure 1 (I)**

**
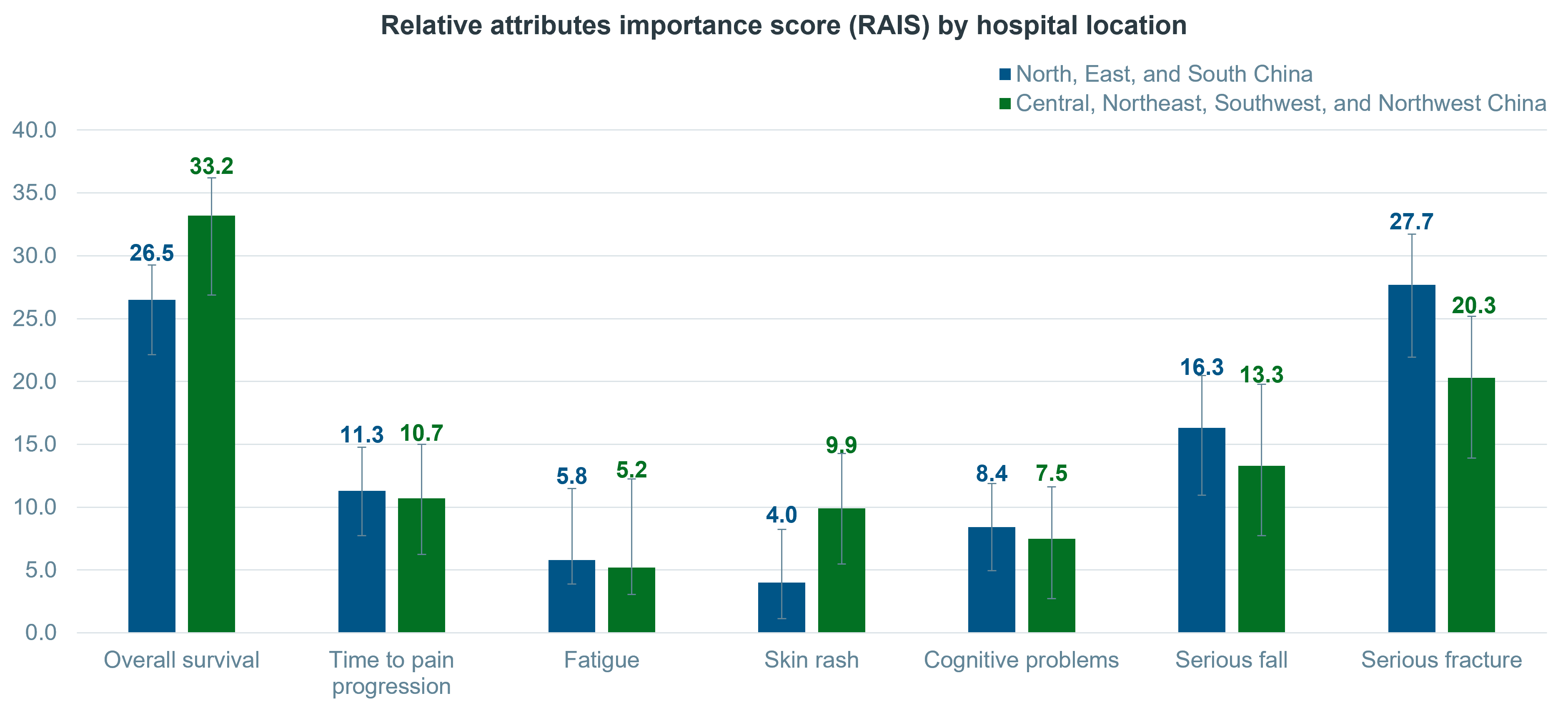
**

**eFigure 1 (J)**

**
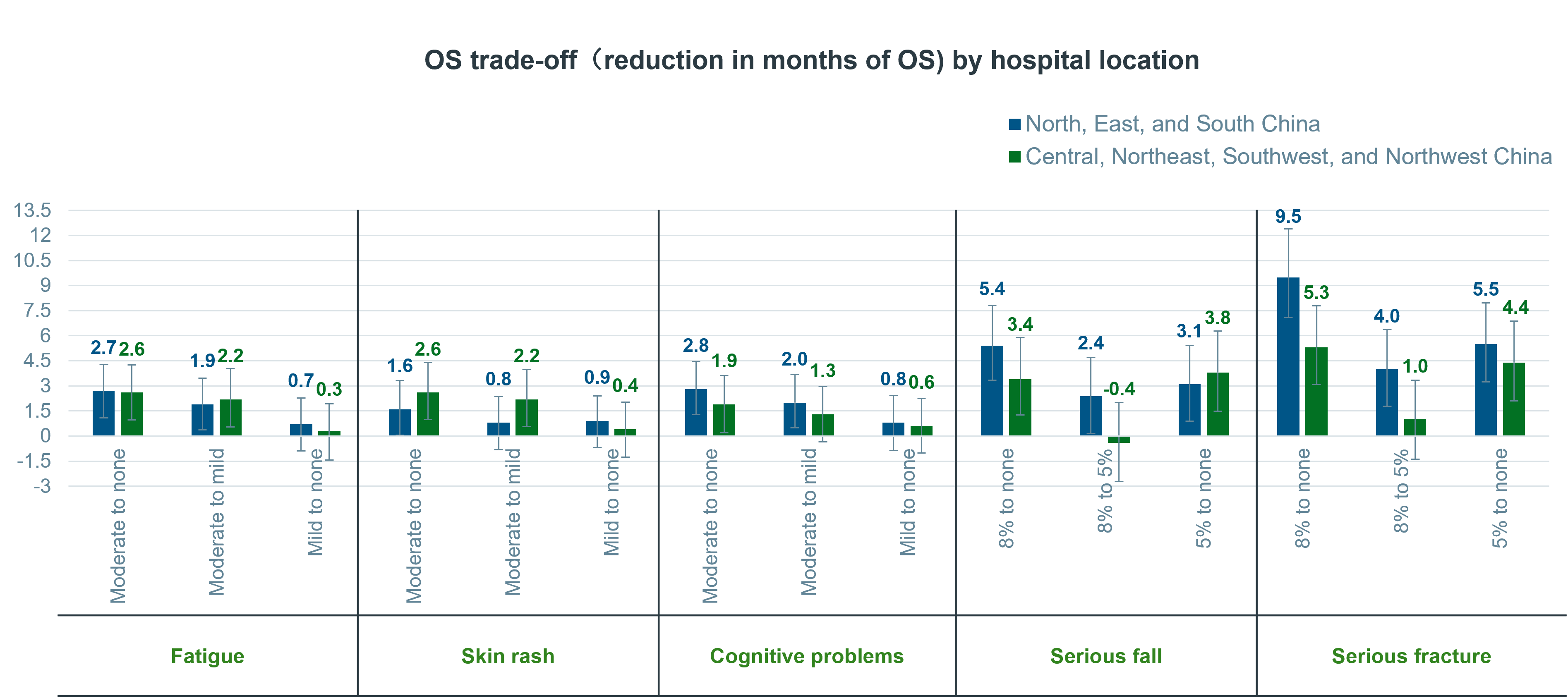
**

**Table S1** **Two efficacy attributes and five safety attributes were covered in the questionnaire, with each attribute containing three levels**


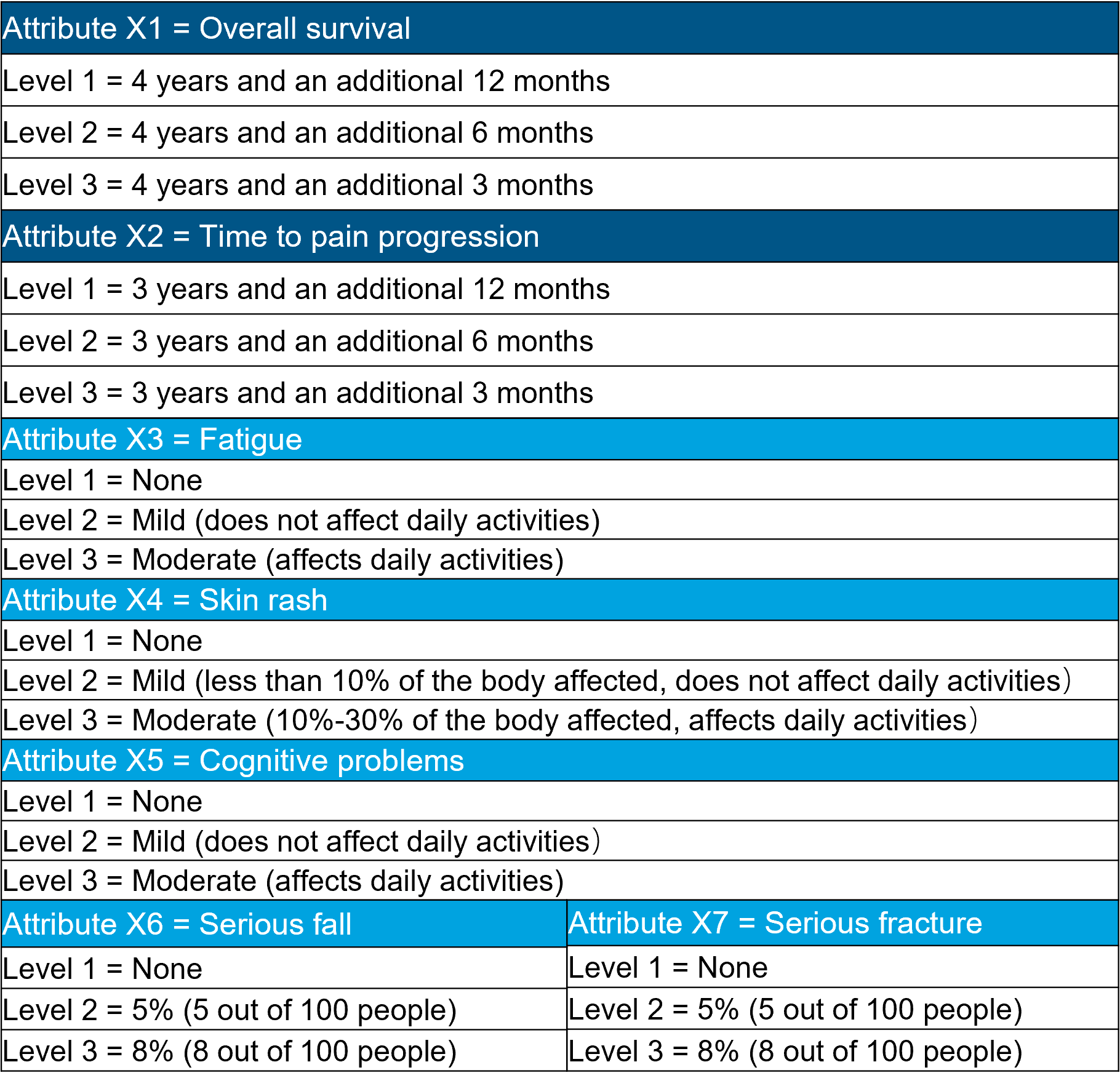


**Table S2 Experience of nmCRPC management of the 80 physicians**

| **Characteristic** | **Value** |
| --- | --- |
| **Patients in which of the following categories were CRPC patients** |  |
| Castrate level of serum testosterone, PSA >2 ng/mL, 3 consecutive increases in PSA 1 week apart by more than 50% from baseline | 48 (60.0) |
| Castrate level of serum testosterone, PSA >1 ng/mL, 2 consecutive increases in PSA 1 week apart | 3 (3.8) |
| Castrate level of serum testosterone, PSA >2 ng/mL, 2 consecutive increases in PSA 1 week apart | 9 (11.3) |
| Castrate level of serum testosterone, 2 consecutive increases in PSA by more than 50% from baseline | 15 (18.8) |
| Castrate level of serum testosterone, at least 2 consecutive increases in PSA 1 week apart | 5 (6.3) |
| **Number of patients with nmCRPC treated in the past year** | 35 [33.03] |
| Number of patients with nmCRPC treated in the past year ≤20 | 43 (53.8) |
| Number of patients with nmCRPC treated in the past year >20 | 37 (46.3) |
| **ADT most frequently prescribed to patients** |  |
| Goserelin acetate | 51 (63.8) |
| Leuprolide acetate | 26 (32.5) |
| Triptorelin | 3 (3.8) |
| **Three drugs most frequently prescribed to patients in addition to ADT, n (%)** |  |
| Abiraterone acetate + prednisone/prednisolone | 65 (81.3) |
| Bicalutamide | 52 (65.0) |
| Enzalutamide | 44 (55.0) |
| Apalutamide | 32 (40.0) |
| Flutamide | 16 (20.0) |
| Docetaxel | 16 (20.0) |
| Darolutamide | 12 (15.0) |
| Olaparib/niraparib | 3 (3.8) |

Data were shown as **n (%) or** mean [SD].

CRPC = castration-resistant prostate cancer； nmCRPC = nonmetastatic castration-resistant prostate cancer
